# A self administered reliable questionnaire to assess lower bowel symptoms

**DOI:** 10.1186/1471-230X-8-8

**Published:** 2008-03-01

**Authors:** Barbara-Ann Adelstein, Les Irwig, Petra Macaskill, Peter H Katelaris, David B Jones, Les Bokey

**Affiliations:** 1 Screening and Test Evaluation Program (STEP), School of Public Health, University of Sydney, Australia; 2 Gastroenterology Dept, Concord Hospital, University of Sydney, Australia; 3 Department of Colon and Rectal Surgery, Concord Hospital, University of Sydney, Australia

## Abstract

**Background:**

Bowel symptoms are considered indicators of the presence of colorectal cancer and other bowel diseases. Self administered questionnaires that elicit information about lower bowel symptoms have not been assessed for reliability, although this has been done for upper bowel symptoms. Our aim was to develop a self administered questionnaire for eliciting the presence, nature and severity of lower bowel symptoms potentially related to colorectal cancer, and assess its reliability.

**Methods:**

Immediately before consulting a gastroenterologist or colorectal surgeon, 263 patients likely to have a colonoscopy completed the questionnaire. Reliability was assessed in two ways: by assessing agreement between patient responses and (a) responses given by the doctor at the consultation; and (b) responses given by patients two weeks later.

**Results:**

There was more than 75% agreement for 78% of the questions for the patient-doctor comparison and for 92% of the questions for the patient-patient comparison. Agreement for the length of time a symptom was present, its severity, duration, frequency of occurrence and whether or not medical consultation had been sought, all had agreement of greater than 70%. Over all questions, the chance corrected agreement for the patient-doctor comparison had a median kappa of 65% (which represents substantial agreement), interquartile range 57–72%. The patient-patient comparison also showed substantial agreement with a median kappa of 75%, interquartile range 68–81%.

**Conclusion:**

This self administered questionnaire about lower bowel symptoms is a useful way of eliciting details of bowel symptoms. It is a reliable instrument that is acceptable to patients and easily completed. Its use could guide the clinical consultation, allowing a more efficient, comprehensive and useful interaction, ensuring that all symptoms are assessed. It will also be a useful tool in research studies on bowel symptoms and their predictive value for colorectal cancer and other diseases. Studies assessing whether bowel symptoms predict the presence of colorectal cancer should provide estimates of the reliability of the symptom elicitation.

## Background

Bowel symptoms are considered indicators of the presence of colorectal cancer and other bowel diseases. However, there is a dearth of reliable self administered questionnaires that elicit information about lower bowel symptoms.

Details of bowel symptoms are usually obtained from patients as part of a face-to-face clinical consultation. However, a self administered questionnaire may be an efficient way of eliciting such information for clinical care and screening programs. Self reported questionnaires about bowel symptoms have been used successfully to assess patients with upper gastrointestinal disease, to discriminate between organic and functional bowel disease and to assess faecal incontinence and constipation [1-8]. The reliability of some of these questionnaires has also been assessed.

Questionnaires to assess lower bowel symptoms relevant to colorectal cancer have also been developed. However, the importance of symptoms is disputed. Some papers suggest that specific symptoms may be useful to predict colorectal cancer [9-16], while others have found no association for any symptoms [17-21]. One of the reasons for this disparity in results may be the quality of symptom elicitation; yet we could find little research assessing the reliability of the questionnaire items used.

The aim of this study is to develop and assess the reliability of an accessible and acceptable questionnaire about bowel symptoms with particular relevance to colorectal cancer, which can be used clinically and for research.

## Methods

### Questionnaire design

Content and face validity was achieved by basing the questionnaire on literature review to determine question content, with emphasis placed on symptoms that may have predictive value for colorectal cancer, and on established questionnaires [3,5,22,23]. Gastroenterologists and colorectal surgeons were asked to comment on the relevance and clarity of the questions, and an iterative process with these specialists was undertaken to decide which symptoms to include and how to word the questions.

Accessibility (understanding of the questions, and that the questions ask what they purport to ask) was achieved by interviewing 20 patients who completed the initial draft of the questionnaire while waiting to see their gastroenterologist or surgeon in consultation. Changes suggested by this process were included in the questionnaire, and the process repeated until no further problems were found.

Readability of the questionnaire was assessed from Microsoft Word 2003 using the Flesch reading score and the Flesch-Kincaid reading level. The questionnaire has a Flesch-Kincaid Reading Grade Level of 4.6 and a Flesch Reading Ease score of 78.9. These scores are based on the average number of syllables per word and words per sentence. The Flesch-Kincaid Grade Level score rates text based on the U.S. high school grade level system (i.e. a score of 4.0 would mean a 4th grader should be able to comprehend the text). The Flesch Reading Ease score is based on a 100 point scale; the higher the score, the easier it is to comprehend. "Plain English" has a score of 65, which has an average sentence length of 15 to 20 words, and an average word of two syllables [24].

**Figure 1:**
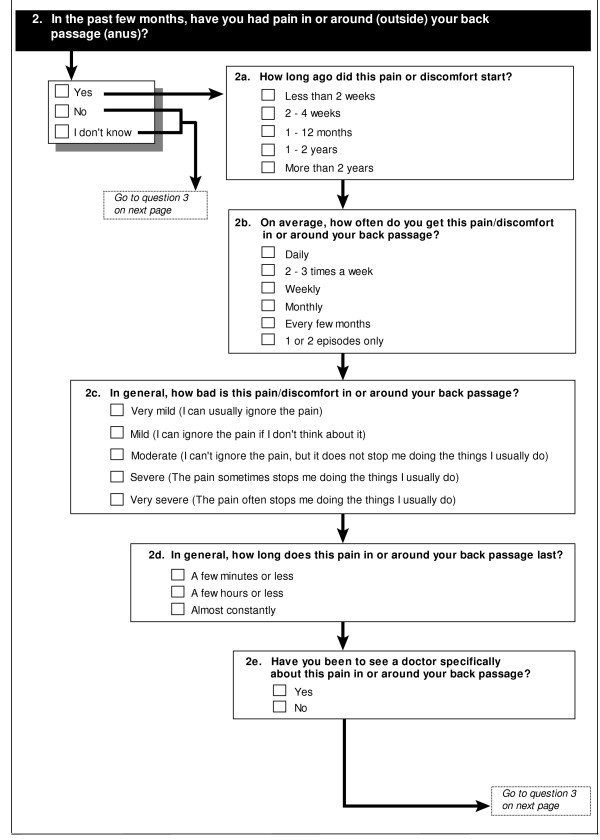
Example of a page from the questionnaire.

The questionnaire we developed captures the information about the presence or absence of symptoms, their characteristics including severity, duration and timing, and whether the symptom alone was regarded as serious enough to prompt seeking medical advice. The questions were presented in a flow diagram format, with connecting arrows. The questionnaire was presented in a stapled booklet format, and consisted of 10 pages of questions, and a cover page for the participant's name and instructions for completing the questionnaire. There were 12 questions about bowel symptoms. These had an initial question asking about the presence of the symptom. If the symptom was present, the participant was directed to further subquestions about detail of that symptom. An example of a question is shown in Figure 1. The full questionnaire is shown in the attached file [see Additional file 1]. The questionnaire generally takes less than 15 minutes to complete.

### Assessing agreement

The study was conducted with gastroenterologists and colorectal surgeons at the Concord Repatriation General Hospital, Sydney, Australia. The study was approved by the University of Sydney and Central Sydney Area Health Service (CRGH Zone) Ethics Committees.

Patients attending for consultation were invited to participate. Those patients with predominantly lower gastrointestinal symptoms in the referral letter, who might subsequently be advised to have a colonoscopy, were included. Participants completed the questionnaire in the waiting room immediately prior to their consultation with the doctor. Exclusion criteria were patients younger than 18 years, and insufficient English proficiency to complete the questionnaire.

Although some may assess questionnaire validity by considering the doctors' responses to be the reference standard, such an approach assumes that the doctors are more accurate than patients in determining symptoms. As there is little evidence for this assumption, our approach was to assess the reliability of the questionnaire, which is a measure of the extent to which the same measurements of individuals obtained under different conditions yield similar results [25]. We assessed two components of reliability: reproducibility (the closeness of results obtained in the same test material under a change of observer – inter-observer comparison) by assessing agreement between patients and doctors, and repeatability (the closeness of results obtained in the same test material by the same observer (intra-observer comparison) by assessing agreement within patients.

The study therefore had two components: patient-doctor agreement, and patient-patient agreement. Patient-doctor agreement was assessed in one group of patients by comparing the results obtained from the questionnaire completed by the patient with those from a clinical interview undertaken during the patient's usual consultation with the specialist, immediately after the patient completed the questionnaire. The specialist completed an identical questionnaire, blinded to the patient's response to the self-administered questionnaire.

Patient-patient agreement was assessed in a separate, independent group of patients by comparing the results obtained from the self-administered patient questionnaire completed immediately prior to their consultation with the doctor with those from a second identical questionnaire which was mailed to participants. In the second questionnaire, patients were asked to answer the questions as they remembered their symptoms when they saw their doctor. Where participants did not return the questionnaire, another questionnaire was mailed to them a few weeks later. No incentives were offered for participation.

Testing took place in two phases. Following an initial phase, minor changes were made to the questionnaire. These changes were mainly changing free text answers to tick box responses (based on the answers obtained in the free text form in the initial questionnaire), or to the wording of the options given. For questions that were changed, results are reported only from the second testing phase. Where questions were unchanged between the two questionnaires, the results are reported from both phases.

### Statistical Analysis

Analysis was done using SAS (version 8.02) software. The proportion of responses showing absolute agreement was calculated. The kappa statistic (κ), a measure of agreement that represents the proportion of agreement beyond that expected by chance alone, was also calculated. κ can range between 1 (perfect agreement), and 0 (level of agreement expected by chance alone); κ > 0.80 is considered to reflect almost perfect agreement, κ between 0.61 and 0.80 substantial agreement, 0.41–0.60 moderate agreement, 0.21–0.40 fair agreement, and κ < 0.20 poor agreement [26]. Where the responses to the questions were ordinal, a weighted kappa, using linear weights, has been used.

When assessing agreement for subquestions that were asked if a particular symptom was reported, a category of "symptom not reported" was included in the analysis. Hence, all participants were included to take account of disagreements in the reporting of the presence of the symptom.

McNemar's test has been used to assess whether, for disagreements, there was evidence of a systematic direction for the disagreements. For the patient-doctor study, this assessed whether responses were more commonly reported by patients or by doctors, and for the patient-patient component, this assessed whether responses were more commonly reported on the first or second occasion.

## Results

A total of 263 participants completed the questionnaire (patient-doctor study: n = 122; patient-patient study: n = 141) (see Table 1). For the patient-patient agreement study, there was an 88% response rate for return of the second questionnaire. The second questionnaire was completed an average of 4.2 weeks after the first.

**Table 1:** Study description and numbers participating

	Patient-Doctor Agreement (n)	Patient-Patient Agreement(n)
Phase 1:	61	68
*Changes made to some questions*		
Phase 2:	61	73

Total	122	141

### Patient-Doctor Agreement Study

There were 7 participating specialists: 3 gastroenterologists saw 74 (61%) patients, and 4 colorectal surgeons saw 48 (39%) patients. A total of 122 patients participated. The age range of participants was 21 to 83 years (mean age, 53 years); 58% were male. Thirty percent had a tertiary education (university degree), and a further 20% had a diploma or trade qualification.

Bleeding per rectum, abdominal pain and change in bowel habit were the most frequently reported symptoms (see Table 2). Patients reported up to 12 (range 0 to12) symptoms each (average 9.3, median 5), and doctors reported up to 11 (range 0 to11) symptoms (average 8.5, median 4) per patient.

**Table 2:** Symptom: frequency (ranked by proportion of patients with the symptom in the patient-doctor agreement study)

	**Patient-Doctor Study**				**Patient-Patient Study**	
	
	**Patient**		**Doctor**		**Patient (first questionnaire)**	
	
	**Symptom present**	**%**	**Symptom present**	**%**	**Symptom present**	**%**
**PR Bleeding**	55	45	54	44	53	38
**Abdominal pain**	53	45	48	40	69	50
**Change Bowel Habit**	52	43	50	41	65	46
**Fatigue**	39	34	39	32	47	34
**Urgency**	45	41	37	32	50	37
**Incomplete Evacuation**	42	38	32	27	60	44
Anal pain*	15	26	13	21	19	26
**Weight loss**	18	16	21	17	21	15
**Anal lump**	23	20	14	11	17	12
**Mucus**	14	12	18	15	21	15
**Anaemia**	9	8	7	6	17	12
**Abdominal lump**	7	6	3	2	6	4

Notes: For the patient-doctor agreement study, n = 122 except for missing responses (maximum 8) or where indicated by asterisk; for patient-patient study n = 141, except for missing responses (maximum 7) or where indicated by asterisk.

*for the patient-doctor study: n = 61 (based only on the second phase questionnaire) except for missing responses (maximum 4); for patient-patient study, n = 73 (based only on the second phase questionnaire) except for missing responses (maximum 1)

Comparison of the patient-completed and doctor-completed responses show that in 78% of all questions there was more than 75% agreement (agreement range 65%–96%, median 81%, interquartile range 75–89%). Eight percent (8%) of questions had a κ > 80%, indicating perfect agreement; 58% had a κ between 61 and 80%, indicating substantial agreement; 30% had a κ between 41 and 60%, indicating moderate agreement only 4% (2 questions) had a κ < 40%, indicating fair agreement. The median κ overall was 65% (range 34–89%; interquartile range 57–72%).

Questions were grouped and analysed according to the detail they elicited about the symptom (Table 3). The main question (which elicited information about the presence of a symptom) had a median κ of 59% (interquartile range 57% to 68%) and median agreement of 88% (interquartile range 83%–91%). The duration of symptoms had a median κ of 61% (interquartile range 59% to 64%) and median agreement of 78% (interquartile range 75% to 80%), and the frequency of occurrence had a median κ of 70% (interquartile range 52% to 73%) and median agreement of 75% (interquartile range 74% to 76%). Compared to other symptom detail, severity of a symptom had the highest median κ of 73% (interquartile range 72%–74%), and median agreement of 77% (interquartile range 77% to 78%). Information about other symptom detail is given in Table 3.

**Table 3:** Agreement and κ(%) between question detail categories: Patient- Doctor comparison

	**Presence**			**Time since original onset**			**Severe enough to ** **prompt consultation**		
	
	**n**	**Agree (%)**	**κ(%)**	**n**	**Agree(%)**	**κ(%)**	**n**	**Agree (%)**	**κ(%)**
**PR bleeding**	121	94	89	60	73	73	118	89	86
**Abdominal pain**	117	89	78	57	74	61	110	86	76
**CBH**	120	77	55	57	70	65	111	77	63
**Fatigue**	114	84	68	57	75	71	102	83	66
**Urgency**	105	80	58	51	78	70	99	72	51
**Incomplete Evacuation**	109	74	59	58	79	60	102	74	46
**Anal pain**	57	84	58	57	77	45	55	78	50
**Weight loss**	115	90	68	58	91	67	112	94	75
**Anal lump**	117	87	57	58	86	61	116	88	55
**Mucus**	120	90	64	58	90	69	118	90	55
**Abdominal lump**	119	94	44	58	95	65	118	96	37

**Median**		88	59		79	66		86	55

Note: n refers to the total number of patients and doctors answering this question (for the time since onset, the results are based only on the second phase questionnaire). CBH = Change in bowel habit

When assessing disagreement between the patient and doctor responses, only 4 (out of total of 50 questions) showed evidence of a systematic difference (p < 0.05). Of these, two questions showed a higher response from doctors than patients: 9% more for whether an anal lump was severe enough to prompt consultation (p = 0.01) 12% more for whether or not urgency was severe enough to prompt consultation (p = 0.02). Two questions showed a higher response from patients than doctors: 10% more for how long anal lump had been present (p = 0.03) and 7% more for the presence of mucus (p = 0.02).

### Patient-Patient Agreement Study

Patients were recruited from 9 participating specialists, with 49% of patients attending gastroenterologists and 51% attending colorectal surgeons. A total of 141 patients participated. The age range of patients was 24 to 87 years (mean age of 59 years); 55% percent of the participants were male. Thirty three percent had a tertiary education (university degree), and a further 16% having a diploma or trade qualification.

Abdominal pain, change in bowel habits and a feeling of incomplete evacuation were the symptoms most commonly reported by patients. Rectal bleeding was the fourth most common symptom (Table 2). Patients reported up to 13 (range 0 to 13) symptoms each (average of 11.2 symptoms, median 5) in the first questionnaire, and up to 12 (range 0 to12) symptoms (average 9 symptoms, median 4) in the second questionnaire.

Comparison of the first and second patient responses showed that in 92% of questions there was more than 75% agreement (agreement range 68%–99%, median 86%, interquartile range 81–92%). Thirty four percent (34%) of questions had a κ > 80%, indicating perfect agreement; 52% had a κ between 61 and 80%, indicating substantial agreement; 30% had a κ between 41 and 60%, indicating moderate agreement; 12% had a κ between 21 and 40%, indicating fair agreement only 2% (1 questions) had a κ < 20%, indicating poor agreement.

Questions were grouped and analysed according to the detail they elicited about the symptoms (Table 4). The main question (which elicited information about the presence of a symptom) had a median κ of 72% (interquartile range 65 to 78%) and median agreement of 90% (interquartile range 84 to 93%). The duration of symptoms had a median κ of 77% (interquartile range 75 to 79%) and median agreement of 84% (interquartile range 81 to 86%), frequency of occurrence had a median κ of 81% (interquartile range 80 to 83%) and median agreement of 83% (interquartile range 79 to 83%), and severity of a symptom had a median κ of 71% (interquartile range 65 to 78%), and median agreement of 87% (interquartile range 83 to 91%). Information about other symptom detail is given in Table 4.

**Table 4:** Agreement and κ(%) between question detail categories: Patient- Patient comparison

	**Presence**			**Time since original onset**			**Severe enough to ** **prompt consultation**		
	**n**	**Agree (%)**	**κ(%)**	**n**	**Agree (%)**	**κ(%)**	**n**	**Agree (%)**	**κ(%)**

**PR bleeding**	139	93	86	69	86	91	138	91	87
**Abdominal pain**	136	88	75	69	75	72	134	81	71
**CBH**	140	84	69	71	73	72	139	75	64
**Fatigue**	134	82	63	68	87	74	127	83	69
**Urgency**	130	83	65	69	78	79	125	78	66
**Incomplete Evacuation**	135	84	70	72	75	75	132	82	72
**Anal pain**	71	94	87	71	83	80	71	90	80
**Weight loss**	135	96	86	70	93	88	133	94	80
**Anal lump**	135	89	58	69	94	77	135	89	58
**Mucus**	138	93	74	72	99	87	134	94	76
**Abdominal lump**	135	92	42	69	91	46	134	94	18

Median		90	72		86	94		90	71

Note: n refers to the total number of patients answering this question.

CBH = Change in bowel habit

When assessing disagreement between the first and second patient responses, only 3 (out of total of 50 questions) showed evidence of a systematic difference (p < 0.05). Of these, 2 questions showed a higher response in the first questionnaire: 7% more for the presence of abdominal pain (p = 0.03) and 4% more for whether the pain woke the patient at night (p = 0.04). By contrast, in their second questionnaire, 13% more of patients reported a longer time that a change in bowel habit had been present (p = 0.04).

### Comparison of agreement between patient-doctor and patient-patient completed questionnaires

**Figure 2:**
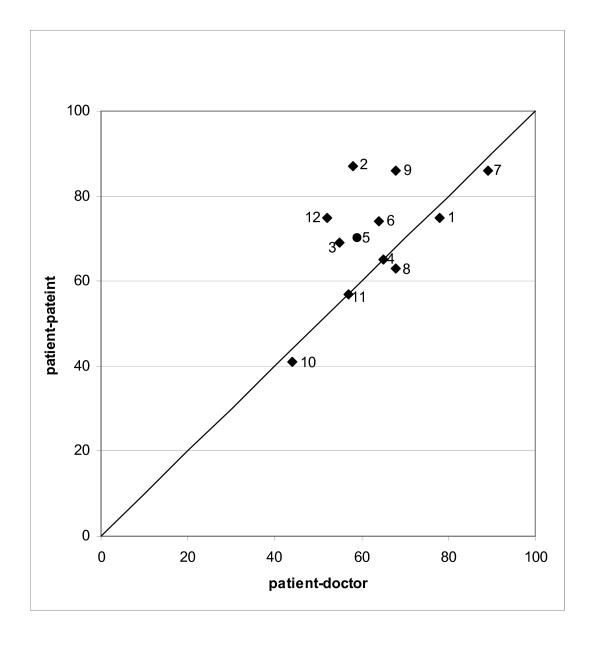
**Scatterplot: Kappa agreement: presence of symptom**. Note: the numbers in the plot refer to the question number. 1 = abdominal pain; 2 = anal pain; 3 = change in bowel habit; 4 = urgency; 5 = incomplete evacuation;; 6 = rectal mucus; 7 = rectal bleeding; 8 = fatigue; 9 = weight loss; 10 = abdominal lump; 11 = anal lump; 12 = anaemia.

The kappa values for patient-patient agreement were consistently higher than those for patient-doctor agreement (Table 4). This is shown graphically in Figure 2 for the main questions (presence of symptoms). The patient-patient kappa values and agreement are also higher than the patient-doctor values for the questions relating to time since onset of the symptom, its frequency, severity and whether it was considered severe enough to prompt medical consultation.

## Discussion

A questionnaire should meet several criteria: it must elicit information of relevance (content validity); the questions must ask what they purport to ask (face validity); it must be accessible; and information obtained must show good agreement between patient and doctor and within patients (patient-patient). Our questionnaire meets these criteria. Compared to general medical history taking and clinical examination, the kappa values and agreement are good [26]. They are similar to those reported for questionnaires applied to upper gastrointestinal disease or faecal incontinence [2,3,5,7,8,22,23].

Our questionnaire was completed by the patient in the waiting room immediately prior to their consultation with the specialist. It might be argued that, at that time, patients are more focussed on their symptoms or are distracted by the imminent consultation. Nevertheless, there is good agreement between the questionnaires completed in the waiting room and those completed several weeks later, so that the timing of administration of the questionnaire does not seem to be an important issue.

Bowel symptom history is usually taken by medical practitioners as part of a face-to-face consultation. We have used the data from the physician interview to assess agreement between this clinical history with that obtained from the patient. However, there is no research to show that the data from the physician's history of symptoms is more accurate than that obtained from patients. Indeed, it has also been shown that patients and doctors may have different perceptions of health problems, and of the importance of these [27]. Health questionnaires completed by patients frequently capture more positive symptoms than are elicited by doctors during consultation [28-30]. This is the case with our questionnaire, with patients reporting on average 1 more symptom than elicited by the specialists.

People presenting with bowel symptoms are often investigated with colonoscopy. There is little high quality evidence to show which symptoms, if any apart from bleeding, improve the diagnostic yield of cancers or precancerous polyps. While some papers suggest that symptoms may be useful to predict colorectal cancer [9-14], others have found no association [17-21]. One recent study in the UK has suggested that a questionnaire can be used to elicit symptoms, and that these symptoms, combined with a weighted numerical score, can be used to predict colorectal cancer [15], and that this combination of symptoms performs better than other symptom groups proposed in cancer referral guidelines [16]. On the other hand, a large study in the USA has found that people with and without bowel symptoms show no difference in rates of colorectal cancer or polyps [31]. With the current drive towards public education about colorectal cancer symptoms it is likely that that many more individuals with minimal symptoms might present for colonoscopy. The costs, both clinical and financial, of performing colonoscopies and the implications for health service provision, both at individual and community levels, are therefore high. It is thus important to assess which symptoms predict and which do not predict the presence of cancer, and an easily applied self administered questionnaire may provide a tool for use in this assessment.

Results in studies about the predictive value of symptoms may differ because of the quality of symptom elicitation. If symptom elicitation is inaccurate or incomplete, the predictive value of the symptoms will be diminished. Misclassification in symptom elicitation between studies may therefore account for the differing study results. To allow adequate interpretation, studies of the predictive validity of symptoms should include estimates of the reliability of the questionnaire, using methods like we have presented.

## Conclusion

Our study shows that this questionnaire is reliable means of assessing bowel symptoms, and is acceptable to patients. Potential application of the questionnaire includes use as part of the clinical consultation to enhance the consultation and to ensure that all patient symptoms are assessed. One of the strengths of this study is the assessment of the agreement between patients and their doctors. This agreement was good. On average, patients reported one more symptom than reported by the doctor. Use of the questionnaire could therefore facilitate discussion of all patient symptom concerns. Its use could guide the consultation, allowing a more efficient, comprehensive and useful interaction. It may also have use for research, for example to assess the significance and predictive value of symptoms for colorectal cancer, and as part of a bowel cancer screening program to elicit symptoms of potential significance. The questionnaire can be used as a reliable standardised instrument in studies to assess the predictive validity of symptoms for colorectal cancer.

## Competing interests

The author(s) declare that they have no competing interests.

## Authors' contributions

BA and LI developed the core idea and designed the study. BA, PK and LI designed, revised and reviewed the questionnaire. BA collected the data. BA and PM did the statistical analysis. PK, BJ and LB completed some of the doctor questionnaires. All authors interpreted the data and wrote the paper. All authors read and approved the final manuscript.

## Pre-publication history

The pre-publication history for this paper can be accessed here:



## Supplementary Material

Additional file 1 Bowel symptom questionnaire. Copy of the questionnaire used to elicit bowel symptoms Click here for file

## References

[B1] Osterberg A, Graf W, Karlbom U, Pahlman L (1996). Evaluation of a questionnaire in the assessment of patients with faecal incontinence and constipation. Scandinavian Journal of Gastroenterology.

[B2] Talley NJ, Phillips SF, Wiltgen CM, Zinsmeister AR, Melton LJ (1990). Assessment of functional gastrointestinal disease: the bowel disease questionnaire. Mayo Clinic Proceedings.

[B3] Talley NJ, Phillips SF, Melton J, Wiltgen C, Zinsmeister AR (1989). A patient questionnaire to identify bowel disease. Annals of Internal Medicine.

[B4] Frank L, Kleinman L, Farup C, Taylor L, Miner P (1999). Psychometric validation of a constipation symptom assessment questionnaire. Scandinavian Journal of Gastroenterology.

[B5] Agreus L, Svardsudd K, Nyren O, Tibblin G (1993). Reproducibility and validity of a postal questionnaire. The abdominal symptom study. Scandinavian Journal of Primary Health Care - Supplement.

[B6] Chisholm EM, de Dombal FT, Giles GR (1985). Validation of a self administered questionnaire to elicit gastrointestinal symptoms. British Medical Journal Clinical Research Ed.

[B7] Romero Y, Thistle JL, Longstreth GF, Harmsen WS, Schleck CD, Zinsmeister AR, Pardi DS, Zein CO, Van Dyke CT, Arora AS, Locke GR (2003). A questionnaire for the assessment of biliary symptoms. American Journal of Gastroenterology.

[B8] Reilly WT, Talley NJ, Pemberton JH, Zinsmeister AR (2000). Validation of a questionnaire to assess fecal incontinence and associated risk factors: Fecal Incontinence Questionnaire. Diseases of the Colon & Rectum.

[B9] Fijten GH, Starmans R, Muris JW, Schouten HJ, Blijham GH, Knottnerus JA (1995). Predictive value of signs and symptoms for colorectal cancer in patients with rectal bleeding in general practice.. Fam Pract.

[B10] Neugut AI, Garbowski GC, Waye JD, Forde KA, Treat MR, Tsai JL, Lee WC (1993). Diagnostic yield of colorectal neoplasia with colonoscopy for abdominal pain, change in bowel habits, and rectal bleeding.. Am J Gastroenterol.

[B11] Norrelund N, Norrelund H (1996). Colorectal cancer and polyps in patients aged 40 years and over who consult a GP with rectal bleeding. Family Practice.

[B12] Staniland JR, Ditchburn J, de_Dombal FT (1976). Clinical presentation of diseases of the large bowel. A detailed study of 642 patients.. Gastroenterology.

[B13] Wauters H, Van Casteren V, Buntinx F (2000). Rectal bleeding and colorectal cancer in general practice: diagnostic study. Bmj.

[B14] Curless R, French J, Williams GV, James OF (1994). Comparison of gastrointestinal symptoms in colorectal carcinoma patients and community controls with respect to age. Gut.

[B15] Selvachandran SN, Hodder RJ, Ballal MS, Jones P, Cade D (2002). Prediction of colorectal cancer by a patient consultation questionnaire and scoring system: a prospective study. Lancet.

[B16] Hodder RJ, Ballal M, Selvachandran SN, Cade D (2005). Variations in the evaluation of colorectal cancer risk. Colorectal Disease.

[B17] Farrands PA, O'Regan D, Taylor I (1985). An assessment of occult blood testing to determine which patients with large bowel symptoms require urgent investigation. British Journal of Surgery.

[B18] Jensen J, Kewenter J, Swedenborg J (1993). The correlation of symptoms, occult blood tests, and neoplasms in patients referred for double-contrast barium enema.. Scand J Gastroenterol.

[B19] Mant A, Bokey EL, Chapuis PH, Killingback M, Hughes W, Koorey SG, Cook I, Goulston KJ, Dent OF (1989). Rectal bleeding. Do other symptoms aid in diagnosis?. Dis Colon Rectum.

[B20] Muris JW, Starmans R, Fijten GH, Crebolder HF, Krebber TF, Knottnerus JA (1993). Abdominal pain in general practice. Family Practice.

[B21] Aldridge MC, Sim AJ (1986). Colonoscopy findings in symptomatic patients without X-ray evidence of colonic neoplasms. Lancet.

[B22] Talley NJ, Boyce PM, Owen BK, Newman P, Paterson KJ (1995). Initial validation of a bowel symptom questionnaire and measurement of chronic gastrointestinal symptoms in Australians. Australian & New Zealand Journal of Medicine.

[B23] Talley NJ, Jones M (1998). Self-reported rectal bleeding in a United States community: prevalence, risk factors, and health care seeking. American Journal of Gastroenterology.

[B24] Flesch RF (1974). The art of readable writing.

[B25] Everitt BS (1998). The Cambridge Dictionary of Statistics.

[B26] Sackett DL, Haynes RB, Guyatt GH, Tugwell P (1991). Clinical Epidemiology:  A Basic Science for Clinical Medicine.

[B27] Scheuer E, Steurer J, Buddeberg C (2002). Predictors of differences in symptom perception of older patients and their doctors. Family Practice.

[B28] Sprangers MA, Aaronson NK (1992). The role of health care providers and significant others in evaluating the quality of life of patients with chronic disease: a review. [Review] [109 refs]. Journal of Clinical Epidemiology.

[B29] Fromme EK, Eilers KM, Mori M, Hsieh YC, Beer TM (2004). How accurate is clinician reporting of chemotherapy adverse effects? A comparison with patient-reported symptoms from the Quality-of-Life Questionnaire C30. Journal of Clinical Oncology.

[B30] Justice AC, Chang CH, Rabeneck L, Zackin R (2001). Clinical importance of provider-reported HIV symptoms compared with patient-report. Medical Care.

[B31] Lieberman DA, de Garmo PL, Fleischer DE, Eisen GM, Chan BK, Helfand M (2000). Colonic neoplasia in patients with nonspecific GI symptoms. Gastrointestinal Endoscopy.

