# Supplementary material for: A self administered reliable questionnaire to assess lower bowel symptoms
Source: BMC Gastroenterol. 2008 Mar 1;8:8. doi: 10.1186/1471-230X-8-8 (PMC2311315; doi:10.1186/1471-230X-8-8)
Supplement: Additional file 1 — Bowel symptom questionnaire. Copy of the questionnaire used to elicit bowel symptoms [file 1471-230X-8-8-S1.pdf]

Name: ______________________

Date: ______________________

The CRISP Study

Colonoscopy Research Into Symptom Prediction

Thank you for participating in this important research.

The arrows will guide you to the appropriate boxes. All you need to do is tick the answers that best describe you, or write in the space provided.

Please answer all questions. Please tick only 1 answer for each question. If you are not sure about an answer, please tick or write your best guess.

All responses will be kept confidential.

If you have any questions about the survey, contact Dr Barbara-Ann Adelstein, on 9036 9042.

The questions begin over the page.

for Office use:

Specialist's name: ______________________

Completed by: Patient ☐ Doctor ☐

Old Patient ☐ New patient ☐

Survey No: ☐☐☐☐ ☐☐☐☐☐

Group: 1 2 3 Cppy ☐

School of Public Health
The University of Sydney

SCREENING & TEST EVALUATION PROGRAM

1. In the past few months, have you had pain or discomfort in your stomach (abdomen)? Do not include cramps or pain with menstrual periods, or pain in your chest)

☐ Yes
☐ No
☐ I don't know

Go to question 2 on next page

1a. How long ago did this pain or discomfort start?

☐ Less than 2 weeks
☐ 2 - 4 weeks
☐ 1 - 12 months
☐ 1 - 2 years
☐ More than 2 years

1b. On average, how often do you get this pain/discomfort?

☐ Daily
☐ 2 - 3 times a week
☐ Weekly
☐ Monthly
☐ Every few months
☐ Occasionally (1 or 2 episodes only)

1c. In general, how bad is this pain or discomfort?

☐ Very mild (I can usually ignore the pain)
☐ Mild (I can ignore the pain if I don't think about it)
☐ Moderate (I can't ignore the pain, but it does not stop me doing the things I usually do)
☐ Severe (The pain sometimes stops me doing the things I usually do)
☐ Very severe (The pain often stops me doing the things I usually do)

1d. In general, how long does this pain last?

☐ A few minutes or less
☐ A few hours or less
☐ Almost constantly

1e. In general, does this pain wake you at night?

☐ Yes
☐ No

1f. In general, when does this pain occur?

☐ Mainly during the day
☐ Mainly during the night
☐ Anytime during the day or night

1g. Circle the letter/s (in the picture below) that show/s where the pain or discomfort usually is.

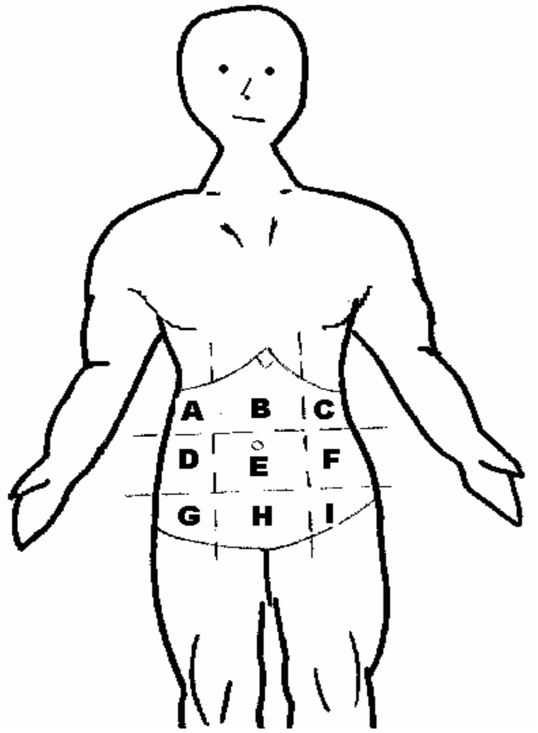

1h. Have you been to see a doctor specifically about this pain/discomfort?

☐ Yes
☐ No

Go to question 2 on next page

2. In the past few months, have you had pain in or around (outside) your back passage (anus)?

- [ ] Yes → go to 2a
- [ ] No
- [ ] I don't know

Go to question 3 on next page

2a. How long ago did this pain or discomfort start?

- [ ] Less than 2 weeks
- [ ] 2 - 4 weeks
- [ ] 1 - 12 months
- [ ] 1 - 2 years
- [ ] More than 2 years

2b. On average, how often do you get this pain/discomfort in or around your back passage?

- [ ] Daily
- [ ] 2 - 3 times a week
- [ ] Weekly
- [ ] Monthly
- [ ] Every few months
- [ ] 1 or 2 episodes only

2c. In general, how bad is this pain/discomfort in or around your back passage?

- [ ] Very mild (I can usually ignore the pain)
- [ ] Mild (I can ignore the pain if I don't think about it)
- [ ] Moderate (I can't ignore the pain, but it does not stop me doing the things I usually do)
- [ ] Severe (The pain sometimes stops me doing the things I usually do)
- [ ] Very severe (The pain often stops me doing the things I usually do)

2d. In general, how long does this pain in or around your back passage last?

- [ ] A few minutes or less
- [ ] A few hours or less
- [ ] Almost constantly

2e. Have you been to see a doctor specifically about this pain in or around your back passage?

- [ ] Yes
- [ ] No

Go to question 3 on next page

3. Have you had a change in your bowel habits (passing stool)? (Diarrhoea, constipation or change from usual)

- ☐ Yes → go to 3a
- ☐ No → go to question 4
- ☐ I don't know → go to question 4

3a. How long ago did this change start?

- ☐ Less than 2 weeks
- ☐ 2 - 4 weeks
- ☐ 1 - 12 months
- ☐ 1 - 2 years
- ☐ More than 2 years

3b. What is this change?: *(You can tick more than 1 answer for this question)*

- ☐ Looser bowel motions (stools) than usual
- ☐ More frequent bowel motions (stools) than usual
- ☐ Harder bowel motions (stools) than usual
- ☐ Less frequent bowel motions (stools) than usual
- ☐ Varies between looser and harder or more and less frequent

3c. Have you been to see a doctor specifically about this change?

- ☐ Yes
- ☐ No

4. In the past few months, have you had to "rush" to open your bowels (pass stool)?

- ☐ Yes → go to 4a
- ☐ No → *Go to question 5 on next page*
- ☐ I don't know → *Go to question 5 on next page*

4a. How long ago did this need to "rush" start?

- ☐ Less than 2 weeks
- ☐ 2 - 4 weeks
- ☐ 1 - 12 months
- ☐ 1 - 2 years
- ☐ More than 2 years

4b. Have you been to see a doctor specifically about this need to rush?

- ☐ Yes
- ☐ No

Go to question 5 on next page

5. In the past few months, have you felt as if you have not emptied your bowels satisfactorily (incomplete evacuation) after you have gone to the toilet?

- ☐ Yes
- ☐ No
- ☐ I don't know

5a. How long ago did this feeling start?

- ☐ Less than 2 weeks
- ☐ 2 - 4 weeks
- ☐ 1 - 12 months
- ☐ 1 - 2 years
- ☐ More than 2 years

5b. Have you been to see a doctor specifically about this feeling?

- ☐ Yes
- ☐ No

6. In the past few months, have you noticed mucus (white or green jelly-like material) in your bowel motions?

- ☐ Yes
- ☐ No
- ☐ I don't know

Go to question 7 on next page

6a. How long ago did this mucus (jelly-like material) start?

- ☐ Less than 2 weeks
- ☐ 2 - 4 weeks
- ☐ 1 - 12 months
- ☐ 1 - 2 years
- ☐ More than 2 years

6b. Have you been to see a doctor specifically about this mucus (jelly-like material)?

- ☐ Yes
- ☐ No

Go to question 7 on next page

7. In the past few months, have you noticed any bleeding from your back passage (anus) when you go to the toilet (open your bowels)?

- ☐ Yes → go to 7a
- ☐ No
- ☐ I don't know
- ☐ I don't look

Go to question 8 on next page

7a. How long ago did this bleeding start?

- ☐ Less than 2 weeks
- ☐ 2 - 4 weeks
- ☐ 1 - 12 months
- ☐ 1 - 2 years
- ☐ More than 2 years

7b. Is the blood:

- ☐ Fresh blood - bright red
- ☐ Old blood - dark red
- ☐ Both fresh (bright red) and old (dark red)
- ☐ I don't know

7c. On average, how often do have this bleeding?

- ☐ Daily
- ☐ 2 - 3 times a week
- ☐ Weekly
- ☐ Monthly
- ☐ Every few months
- ☐ Occasionally (1 or 2 episodes only)

7d. On average, is the bleeding (each time):

- ☐ A smear or drop on the toilet paper only
- ☐ A small amount (approximately less than a teaspoon)
- ☐ A moderate amount (approximately less than a cup)
- ☐ A large amount (approximately more than a cup)
- ☐ I don't know

7e. Is the blood: *(You can tick more than 1 answer for this question)*

- ☐ On the toilet paper
- ☐ In the toilet bowl
- ☐ Mixed with stool (bowel motion)
- ☐ Coating the stool (bowel motion)
- ☐ I don't know

7f. Have you been to see a doctor specifically about this bleeding?

- ☐ Yes
- ☐ No

Go to question 8 on next page

8. In the past few months, have you been more tired (fatigued) than usual?

- ☐ Yes
- ☐ No
- ☐ I don't know

8a. How long ago did this feeling start?

- ☐ Less than 2 weeks
- ☐ 2 - 4 weeks
- ☐ 1 - 12 months
- ☐ 1 - 2 years
- ☐ More than 2 years

8b. Have you been to see a doctor specifically about this tiredness (fatigue)?

- ☐ Yes
- ☐ No

9. In the past few months, have you lost weight (without dieting)?

- ☐ Yes
- ☐ No
- ☐ I don't know

Go to question 10 on next page

9a. How long ago did this weight loss start?

- ☐ Less than 2 weeks
- ☐ 2 - 4 weeks
- ☐ 1 - 12 months
- ☐ 1 - 2 years
- ☐ More than 2 years

9b. How much weight have you lost during this time?
(Please estimate if you do not know exactly)

☐☐ kgs OR ☐☐ lbs

9c. Have you been to see a doctor specifically about this weight loss?

- ☐ Yes
- ☐ No

Go to question 10 on next page

10. In the past few months, have you felt a lump in your stomach (abdomen)?

- ☐ Yes → go to 10a
- ☐ No → go to 11
- ☐ I don't know → go to 11

10a. How long ago did you first notice this lump in your stomach (abdomen)?

- ☐ Less than 2 weeks
- ☐ 2 - 4 weeks
- ☐ 1 - 12 months
- ☐ 1 - 2 years
- ☐ More than 2 years

10b. Have you been to see a doctor specifically about this lump in your stomach (abdomen)?

- ☐ Yes
- ☐ No

11. In the past few months, have you felt a lump around your back passage (anus) when you wipe yourself?

- ☐ Yes → go to 11a
- ☐ No
- ☐ I don't know

Go to question 12 on next page

11a. How long ago did you first feel this lump around your back passage (anus)?

- ☐ Less than 2 weeks
- ☐ 2 - 4 weeks
- ☐ 1 - 12 months
- ☐ 1 - 2 years
- ☐ More than 2 years

11b. Have you been to see a doctor specifically about this lump around your back passage (anus)?

- ☐ Yes
- ☐ No

Go to question 12 on next page

12. In the past few months, have you been told that you are anaemic (have a low blood count)?

- ☐ Yes
- ☐ No
- ☐ I don't know

12a. How long have you been anaemic (had a low blood count?

- ☐ Less than 2 weeks
- ☐ 2 - 4 weeks
- ☐ 1 - 12 months
- ☐ 1 - 2 years
- ☐ More than 2 years

13. Have you ever had a bowel condition?

- ☐ Yes
- ☐ No
- ☐ I don't know

- ☐ Polyps in your bowel
- ☐ Bowel / colon cancer
- ☐ Rectal cancer
- ☐ Ulcerative colitis
- ☐ Crohn's disease
- ☐ Piles / haemorrhoids
- ☐ Irritable Bowel Syndrome
- ☐ Anal Fissure
- ☐ Diverticulitis

14. Have you ever had a colonoscopy?

- ☐ Yes
- ☐ No
- ☐ I don't know

Go to question 15 on next page

14a. How many colonoscopies have you had?

- ☐ 1 only
- ☐ More than 1

14b. In what year was your most recent colonoscopy done?

☐☐☐☐

Go to question 15 on next page

15. Have you ever had surgery to remove part of your bowel?

- ☐ Yes
- ☐ No
- ☐ I don't know

16. Has anyone in your family (blood relative) had bowel (colon or rectal) cancer?

- ☐ Yes
- ☐ No
- ☐ I don't know

16a. Who?

Who?	Age at time of diagnosis
☐ Mother	☐☐ years old
☐ Father	☐☐ years old
☐ Sister	☐☐ years old
☐ Sister (second)	☐☐ years old
☐ Brother	☐☐ years old
☐ Brother (second)	☐☐ years old
☐ Child	☐☐ years old
☐ Child (second)	☐☐ years old
☐ Grandparent	☐☐ years old
☐ Grandparent (second)	☐☐ years old
☐ Aunt	☐☐ years old
☐ Aunt (second)	☐☐ years old
☐ Uncle	☐☐ years old
☐ Uncle (second)	☐☐ years old
☐ Niece/nephew	☐☐ years old
☐ Niece/nephew (second)	☐☐ years old
☐ Other ________	☐☐ years old

Now we would like to ask some questions about you

17. Compared to other people of your age and sex, how seriously do you think you take any symptoms?

- ☐ A lot less seriously than other people
- ☐ Less seriously than other people
- ☐ About the same as other people
- ☐ More seriously than other people
- ☐ A lot more seriously than other people

18. Are you:

- ☐ Male
- ☐ Female

Go to question 19 on next page

19. How old are you?

☐☐ years

20. What is the highest level of education you have completed?

- ☐ No formal education
- ☐ Primary school only
- ☐ Some years at high school
- ☐ Trade certificate
- ☐ School / Intermediate certificate
- ☐ High school certificate / Matriculation
- ☐ Diploma / TAFE qualification
- ☐ University graduate

21. What language do you speak most often at home?

- ☐ English
- ☐ Other (please specify) ______________________________

22. In what country were you born?

- ☐ Australia
- ☐ Other (please specify) ______________________________

23. What is the postcode of the suburb where you usually live?

☐☐☐☐

THANK YOU FOR COMPLETING THIS QUESTIONNAIRE

Please check you have answered all 23 questions and have signed the consent form
